# Influence of sound and light combined conditions in urban environments on residents’ tolerance limits in pre sleep state

**DOI:** 10.3389/fpsyg.2023.1102761

**Published:** 2023-05-15

**Authors:** Yue Yu, Danya Feng, Xin Zhang, Jian Kang

**Affiliations:** ^1^Department of Architecture, School of Architecture, Tsinghua University, Beijing, China; ^2^School of Architecture, South China University of Technology, Guangzhou, China; ^3^School of Architecture, Tsinghua University, Beijing, Beijing, China; ^4^Institute for Environmental Design and Engineering, Bartlett Faculty of the Built Environment, University College London, London, England, United Kingdom

**Keywords:** sound–light interaction, noise pollution, obtrusive light, tolerance limits, pre-sleep state

## Abstract

To determine the sound and light combined conditions pollution in urban residential environments at night, this paper comprehensively evaluates cross-visual and auditory sensory channels in the laboratory. Experimental variables include extremum and gradient, and the working state of the participants was determined and verified. A subjective evaluation experiment on 18 combined conditions was carried out by synthesizing real-world data. Results from the sound and light combined conditions experiment show that there are significant differences in the tolerance limits of participants to different content sound variables (*p* = 0.000 < 0.05, *p* = 0.033 < 0.05, *p* = 0.002 < 0.05). Among them, the traffic noise (*p* = 0.000 < 0.05) has the greatest impact on the tolerance limits of people, followed by birdsong (*p* = 0.033 < 0.05) and human voice (*p* = 0.002 < 0.05). There is no difference in the tolerance limits of light pollution (*p* = 0.288 > 0.05, *p* = 0.122 > 0.05, *p* = 0.146 > 0.05) at different color temperatures. The tolerance limits of participants will not be reduced due to the superposition of two interference variables: sound pollution and light pollution. Adding light pollution to sound pollution can increase the tolerance limits of participants, while adding sound pollution to light pollution has no significant effect on the tolerance limits. The study also found that adding light with different color temperatures to the human voice can increase participants’ tolerance limit to human voice (1% -2%), indicating that visual elements can change individuals’ perception of sound. In addition, the physiological and psychological differences between participants may affect the performance differences of individual participants in sound and light combined conditions.

## 1. Introduction

Sound and light pollution are environmental problems that affect the quality of human life and pose a serious threat to the natural environment and human health (Sivaramanan, 2015). With the rapid development of economy, the areas affected by the sound and light pollution caused by night economic activities (such as road transportation and commercial activities) are expanding, and the impact of strong light on the urban residential environment is becoming more and more significant (Falchi et al., 2011). Research shows that environmental noise pollution can lead to diseases including the nervous, digestive and immune systems, and endanger the physical and mental health of residents (Goines and Hagler, 2007; Xiaoxia, 2007). Long term exposure to strong light in the environment will destroy the normal circadian rhythm, which may lead to organic diseases such as breast cancer and cancer, and endanger health (Kerenyia et al., 1990; Pauley, 2004).

Human perception of the environment is not generated by a single and isolated sense. On the contrary, it is an inherent multi-sensory experience, involving the interaction of visual, auditory, olfactory and other sensory stimuli and the modification of the overall consciousness (Meihui et al., 2020). For example, the cross-effect of indoor visual and auditory can improve the productivity of employees in the open office space (Jeon et al., 2022). If the overall feeling of the indoor environment, as well as the sense of vision and hearing, is simply expressed in a positive and negative way, the following hypothesis can be put forward, that is, the interaction between positive or negative vision, hearing and other sensory channels (such as smell) can make the overall feeling of the indoor environment become more positive or more negative (Kang et al., 2016). The existing research makes use of positive auditory perception (such as positive sound scene) to interact with matched and positive visual perception to improve the overall perception (Val et al., 2006; Benfield et al., 2010; Renterghem, 2018; Ren, 2023). However, there is little research on the interaction between negative auditory perception (such as various kinds of noise) and negative visual perception (such as various types of glare). Therefore, it is not clear how to make use of the interaction of noise and glare to reduce the overall negative feelings of the passive night living environment, which is wrapped by various kinds of noise and glare and makes it difficult to sleep.

In response to the above problems, this study simulates the living environment in the laboratory, and discusses the impact of the interaction of various sound–light pollution on the tolerance limit of participants in the pre-sleep state: how does different types and intensities of sound pollution affect the tolerance limit, different color temperatures How the intensity of light pollution affects the tolerance limit and whether the impact of different types of sound–light pollution interactions on the tolerance limit of participants will be reduced by the superposition of two interference variables. 28 participants spent 2 weeks to complete the subjective evaluation and comparative analysis. In the laboratory, the participants reached the tolerance limit by simulating the outdoor sound pollution, light pollution and sound–light pollution interactions under sleep conditions. Orthogonal experimental design was used to select the sound, and the participants were tested one by one with the “tolerance limit” of optical intrusion and interactive intrusion (adding light, sound or adding sound and light) as the key test point. This study discusses the design intervention theory of sound and light environment, so as to greatly reduce the impact on the environment and provide support for creating a healthier urban environment.

## 2. Methods

This subjective evaluation study of sound and light combined conditions is set in an urban residential environment. It investigates the influence on different participants regarding the tolerance limit of another disturbance variable under the state of one tolerance limit. The results are then statistically analyzed.

The experiment was set up in an indoor environment and simulates participants in a pre-sleep state reaching an tolerance limit caused by adding sound, light and adding sound and light. Common illumination of three color temperatures and three types of sound in an urban environment were selected as the disturbing variables.

### 2.1. Experimental environment

**Figure 1:**
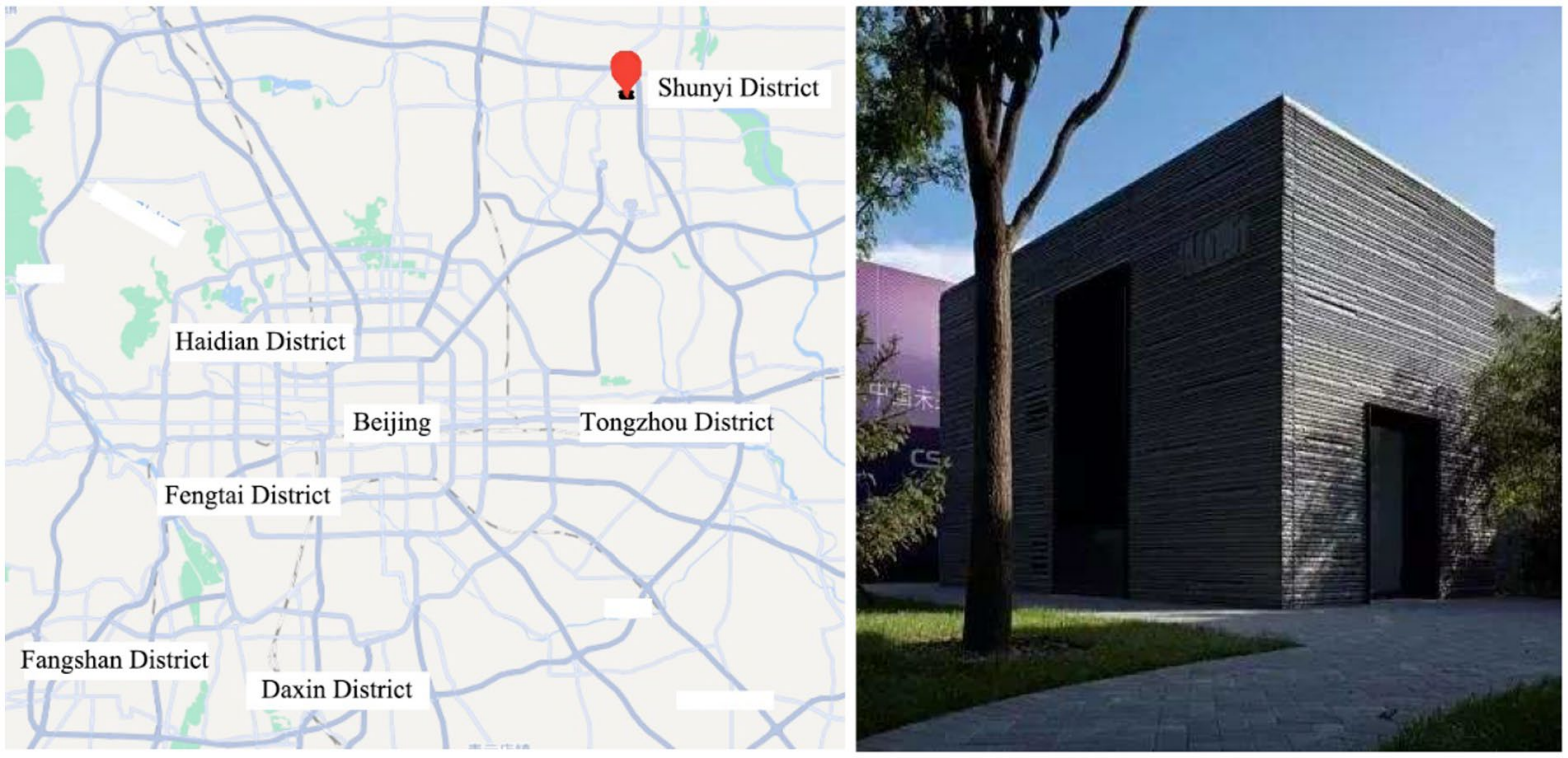
Location of the pre-experiment and the main experiment.

**Figure 2:**
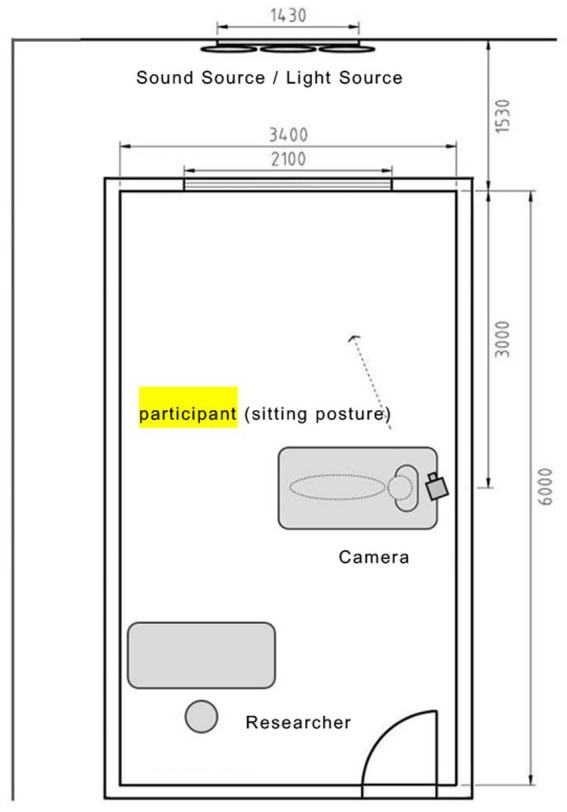
Scene and layout of the experiment.

The experiment was conducted in the No. 1 experimental house (Figure 1) at CSC, Xuhui block 26, Nanjiao Road, Shunyi District, Beijing. The experiment was repeated in a bedroom on the first floor. To simulate real living conditions, the space size was set as 6.0 m × 3.4 m, and the measured reflectance of each surface was 30% for the ground, 88% for the wall, and 88% for the ceiling. Window size was 2.1 m × 1.2 m, and the comprehensive transmittance of frosted glass was 40%. The experiment used a double-layer hollow glass window (5mm Low-E + 12A + 5 mm), which is coated indoors, and reduced the indoor noise to 55 dB. This experimental environment simulated typical indoor space and the experimental conditions of previous studies (Chan, 2021), one experimental bed is set in the experimental area, 3 m away from the window, and the participants face the frosted glass in a lateral recumbent position, the layout is shown in Figure 2. The participants used the side lying posture facing the window to simulate the extreme state of being disturbed by light while sleeping in the urban environment. The bed used in the experiment has a width of 0.9 meters, a length of 3 m, and a height of 0.45 m including the mattress, which is a common single bed size in the bedroom. The participants lie on the side of the bed facing the window, and the vertical distance between the observation position and the window is 3 m, which can ensure that the whole window can be observed in the field of vision. The light source and sound source are set outdoors and controlled *via* WIFI. In order to avoid the impact of hunger on the test results, meals were organized before the experiment. The meal and rest time is 17:00 ~ 18:00, and the experiment time is 18:00 ~ 22:00. During the experiment, all doors and windows remained closed, ambient temperature was controlled at 21°C, and the ambient sound level was 32 dBA. In order to avoid the sound and light stimulation outside the experiment influence the experimental results, the participants were forbidden to use mobile phones, earphones and other listening devices during the preparation and the rest of the experiment. In order to avoid the impact of noise in the experiment on the participants in preparation and during the rest of the experiment, the participants need to use the earplugs during the experiment. A researcher briefly introduced the experimental process to the participants, and one researcher and one participant remained in the laboratory after the experiment started. The experiment was conducted 3 m from the window.

### 2.2. Sound stimulation

**Figure 3:**
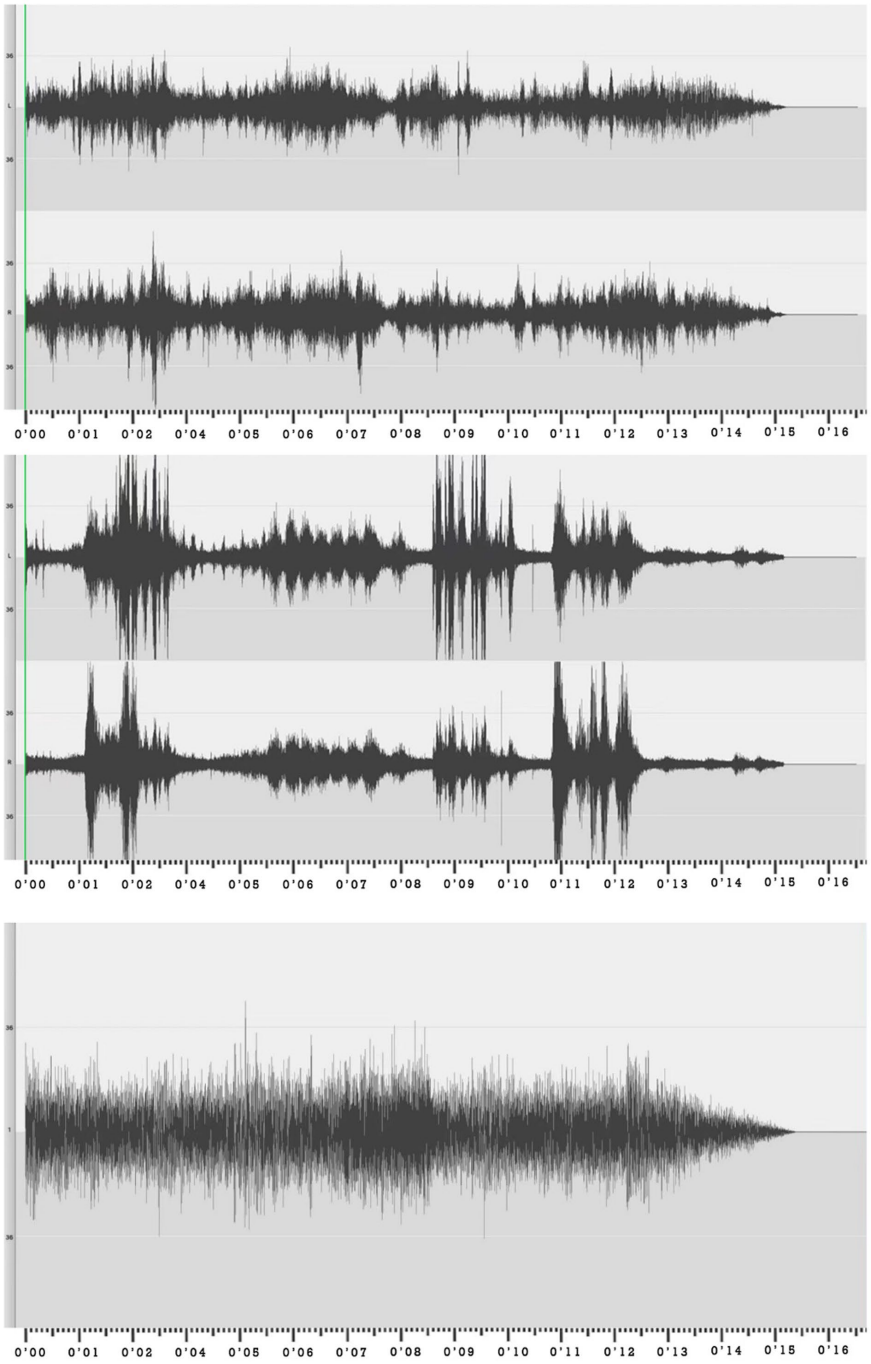
Frequency spectra of birdsong, voice conversation, and traffic noise.

In the experiment, birdsong, voice conversation, and traffic noise were selected as sound variables, corresponding to natural (positive), man-made (neutral), and mechanical (negative) sounds in the urban environment, respectively (Knez, 1995). Using a high-fidelity portable recorder (Figure 3), these sounds were recorded at the Beijing Zoo, the 3w Cafe in Zhongguancun, and the Second Ring Road in North Beijing. The recording device was placed 1.5 m above the ground and it recorded the sounds for 5 min at a time. To avoid a large dynamic range gap in the sample, out-of-range noises were eliminated using audio editing software. Taking traffic noise as an example, a car’s sudden honking and sudden braking sound were eliminated to preserve the smooth spectrum of road tire noise. The average speed of vehicles was 65 km/h, and the proportion of heavy vehicles was 12.5%. The sound variable was played using an outdoor Bluetooth speaker(SA-T35 audio of 80 Hz ~ 18KHz) controlled by an audio control software. The measured data of the gradient of sound variables are shown in Table 1. According to the survey of acoustics assessment of noise annoyance by means of social and socio-acoustic surveys (World Health Organization, 2018 ) the noise level threshold that interferes with sleep is 45 dB at night, so when setting the threshold, this experiment sets 50 dB as the research range.

**Table 1:** Measured gradient data of sound variables.

Sound source	Volume	A-weighted sound pressure level (dB)					
		LA, eq	LA, max	LA, min	LA,10	LA,90	LA,10–LA,90
Traffic noise	Level 1	32.5	34.2	30.6	34.4	29.1	34.4–29.1
	Level 2	35.0	36.8	32.9	37.1	31.3	37.1–31.3
	Level 3	37.5	39.5	35.3	39.7	33.6	39.7–33.6
	Level 4	40.0	42.1	37.6	42.4	35.8	42.4–35.8
	Level 5	42.5	44.7	40.0	45.0	38.0	45.0–38.0
	Level 6	45.0	47.3	42.3	47.7	40.3	47.7–40.3
	Level 7	47.5	50.0	44.7	50.3	42.5	50.3–42.5
	Level 8	50.0	52.6	47.0	53.0	44.7	53.0–44.7
Conversation voice	Level 1	32.5	35.0	30.0	35.7	28.3	35.7–28.3
	Level 2	35.0	37.7	32.3	38.4	30.5	38.4–30.5
	Level 3	37.5	40.4	34.6	41.2	32.6	41.2–32.6
	Level 4	40.0	43.1	36.9	43.9	34.8	43.9–34.8
	Level 5	42.5	45.8	39.3	46.7	37.0	46.7–37.0
	Level 6	45.0	48.5	41.6	49.4	39.2	49.4–39.2
	Level 7	47.5	51.2	43.9	52.2	41.3	52.2–41.3
Bird song	Level 1	32.5	35.3	27.5	36.0	22.1	36.0–22.1
	Level 2	35.0	38.0	29.6	38.8	23.8	38.8–23.8
	Level 3	37.5	40.8	31.8	41.5	25.5	41.5–25.5
	Level 4	40.0	43.5	33.9	44.3	27.2	44.3–27.2
	Level 5	42.5	46.2	36.0	47.1	28.9	47.1–28.9
	Level 6	45.0	48.9	38.1	49.8	30.6	49.8–30.6
	Level 7	47.5	51.6	40.2	52.6	32.3	52.6–32.3

In order to determine the strength and gradient of the test variables, a pre-experiment consisting of 25 people was completed before the formal experiment. Through the “endurance limit” test, the average A sound level tolerance point of the lowest volume was measured to be 33 dB-A, and the maximum was 50 dB-A. The sound level gradient of the formal experiment is adjusted to 2 5 dB-A, total 8 gradient levels.

### 2.3. Light variable

**Figure 4:**
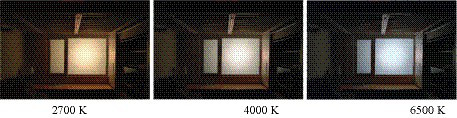
Three kinds of obstacle light used in the experiment.

A lighting investigation of the intermixing of the commercial and residential environment was conducted. This is the most serious obtrusive light in the residential environment in China’s emerging cities. The investigation found that static achromatic light (including low, medium and high color temperature) is the most serious phenomenon of over-standard lighting and it is the main cause of obtrusive light from commercial complexes to surrounding residential environments. Therefore, three typical color temperatures, namely, low, medium and high temperatures (2,700, 4,000, and 6,500 K), were selected as the light variable (Figure 4), and average illuminance was measured on the exterior surface of the window glass through a 12 dot-matrix to quantify the intensity of the light variables.

To make the experimental data and research results more rigorous, the conversion calculation based on the DGInight evaluation index for the average illuminance data of the window facade is needed (Knez, 1995). This is done by setting a camera at the participant’s viewing position to the surroundings to capture each intensity gradient image and record the maximum luminance on the window using a luminometer [color luminance meter (TOPCON BM-7)]. By using “hdrscope” software to process the image taken by the camera and to assist the luminometer in testing the calibration value of screen luminance, the corresponding light source luminance Ls and background luminance Lb of different light variable intensities can be confirmed. According to the position relationship between the observation point and the light source, *ω* and *P* can be obtained, Ω can be obtained after calculation. Based on a literature review (Moghadam et al., 2020), the luminous environment data can be converted into the index of DGInight. According to the DGInight index formula for evaluating indoor and outdoor light intrusion, the result of conversion is shown in Table 2.

**Table 2:** Data conversion calculation of light variable based on DGInight index.

Intension	L (lx)	Ls (nt)	Lb (nt)	Ω	*W*	DGI	DGInight
Level 1	1	0.43	0.04	0.1	0.17	−4.26	15.69
Level 2	2	0.9	0.07			−1.76	17.81
Level 3	3	1.35	0.07			0.51	19.72
Level 4	4	1.78	0.08			1.62	20.66
Level 5	5	2.15	0.08			2.59	21.48
Level 6	7	2.8	0.11			3.14	21.95
Level 7	10	3.66	0.15			3.73	22.45
Level 8	15	5.78	0.19			5.46	23.91
Level 9	20	8.05	0.22			6.73	24.98
Level 10	25	10.97	0.35			7.20	25.37
Level 11	30	13.16	0.46			7.46	25.59
Level 12	40	15.78	0.55			7.94	26.00
Level 13	50	17.87	0.67			8.09	26.12
Level 14	70	19.52	0.69			8.46	26.44
Level 15	90	26.01	1.12			8.72	26.66
Level 16	120	38.4	1.62			9.78	27.56
Level 17	150	50.76	2.12			10.54	28.19
Level 18	190	79.93	3.24			11.80	29.26

*E* is the vertical average illuminance. L_s_ is the luminance of the light source at the horizon. L_b_ is the background luminance. Ω is the Stereo Angle(sr) corrected for the position of the light source at the horizon, and *ω* is the Stereo Angle(sr) of the glare light source.

### 2.4. Experimental procedures

The experiment was set up in an indoor environment, simulating that the participants reached the endurance limit due to the interaction of outdoor sound, light and sound and light when they were asleep. This study includes 3 sound variables and 3 light variables, of which the sound variable has 8 gradients and the light variable has 18 gradients. Therefore, the number of conditions is 3 × 8 × 3 × 18. As there are numerous variable and gradient combinations (3 × 8 × 3 × 18), the experiment was conducted under orthogonal experimental design. The tolerance limits of adding sound, adding light and adding sound and light were selected as the key test points. Table 3 are the “Just intolerable” evaluation points from the endurance evaluation form. First, a single intrusive variable was used to test participants one-by-one on the order of intensity from weak to strong and the test was stopped when participants reported an intolerable level. After recording the endurance limit of each participant to each single variable, the combined sound and light test was carried out, that is, the intrusion of another attribute was added under original endurance limit intensity and the variable intensity was increased gradually until it became intolerable. Then, the tolerance limits of the participants to combined sound and light could be determined. The tolerance limits difference between a single variable and two variable interaction intrusion was compared, and statistical analysis was conducted. Additionally, the difference in tolerance limit between single-variable and double-variable intrusion can be compared and statistically analyzed.

**Table 3:** Tolerance limit evaluation table.

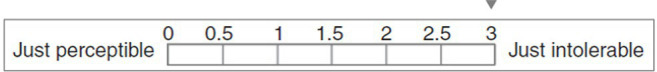

All 28 participants of the main experiment were local college students recruited *via* the Internet or *via* personal contact and were compensated. The participants comprised by 15 females and 13 males, age range from 21 to 30 years old, average age 24 years old (SD = 24; Min = 21; Max = 30), self-reported hearing and vision normal, no mental illness, no pregnancy. The content of this study as it related to the participants passed reviewed by the Tsinghua Institutional Review Board.

Each participant had to complete seven test sessions, of which one is a single-variable test and six are interactive tests. The test simulated a bedroom in which the participants were in a sleep-like state at night and were facing frosted glass in a lateral position. The procedure is as follows:

1. In Group 1, the participants heard sound intrusions (traffic noise, voice conversation, birdsong) three times and saw obtrusive light (2,700, 4000, 6500 K) three times, played one-by-one in order from weak to strong. To avoid fatigue caused by repeated intrusive variables, the duration of each intrusion lasted only 20 s. Then, the participants were asked to evaluate the tolerance limits of the variable at each strength and whether they could accept the current strength. They were asked to report back to the researcher orally and the researcher went on to test the next strength gradient. The variable test was stopped when the intensity reached an intolerable degree for the participants. The researcher recorded the maximum intensity that the participant could tolerate for the variable, that is, the tolerance limits of the participants to the test variable, and the tolerance limits of the participant to each single variable was measured in turn.
2. In Groups 2–4, the tolerance limits of the participants to adding sound was fixed, then, the tolerance limits for the light intrusion variables was determined one-by-one.
3. In Groups 5–7, the tolerance limits of the participants to light intrusion was fixed, then, the tolerance limits for the sound intrusion variables was determined one-by-one. The researcher recorded the tolerance limits of each group for each variable and the interaction intrusion for each participant. At the end of each group of tests, the participants entered the lounge to rest with earplugs, and the experimental assistant notified the next participant to enter the laboratory. The assistant would then ask the next participant to enter the lab.

### 2.5. Data analysis

All statistical analyses were executed using IBM SPSS statistics 26 (IBM Corporation, Somers, New York, NY, United States). (1) Frequency analysis was used to analyze the distribution characteristics and internal structure of the tolerance limits of participants caused by sound–light interaction, so as to obtain an intuitive perceptual understanding, and to determine the analysis method to be used to further analyze the statistical law of variables. (2) One-way analysis of variance (ANOVA) was used to test one (or several independent) dependent variable affected by a single factor. The factors determine whether the difference between the mean values of each level group has statistical significance. We compared the mean values between two groups, known as multiple comparison of mean values between groups. This study tests whether the difference between the participants’ tolerance limits for different content acoustic variables and the mean value of the participants’ tolerance limits level grouping for different color, temperature, and light variables has statistical significance. (3) Chi-square test was used to analyze the correlation between gender differences and tolerance limits under the influence of sound and single photometry. The correlation and significance between the gender of the participants and their tolerance limits under the influence of 2,700 K single metering, 4,000 K single metering, and 6,500 K single metering, as well as their tolerance limits under the influence of traffic, birdsong, and human voice, were calculated. (4) Two-way ANOVA was used to test the main effects of sound and light intrusion and their interaction on the tolerance limits of participants. Moreover, age and gender were regulated as covariates. By investigating the estimated marginal values of single light metering and three traffic sounds and three color temperatures, gender, age, and the estimated values of three single light metering and three traffic tones, pairwise comparison and multivariable univariate tests were used to obtain the individual differences of participants. (5) Single-sample *t*-test was used to study the influence of sound and light combined conditions on individual differences of participants.

## 3. Results

### 3.1. Single-variable test results of sound pollution

The ANOVA and homogeneity of variance test analysis of the results of the single-variable endurance test showed that there was a significant difference in the tolerance limits of the participants to different content sound variables (*p* = 0.000 < 0.05, *p* = 0.033 < 0.05, *p* = 0.002 < 0.05). The results of ANOVA analysis showed that there was a significant difference in the tolerance limits of traffic noise (*p* = 0.000 < 0.05), birdsong (*p* = 0.033 < 0.05), and conversation sound, (*p* = 0.002 < 0.05). The effects of different sound intrusions on human tolerance from low to high are traffic noise, birdsong, and conversation (see Table 4). This result differs from that obtained by Meihui Ba and Jian (Knez, 1995), who studied the comfort, preference, familiarity, and loudness of these three sounds in the laboratory. The results show that natural sound was positively evaluated in terms of comfort and sound preference, while mechanical sound was negatively evaluated. This means that the participants had the best perception of bird calls and the worst perception of traffic noise. However, the study did not involve the evaluation of tolerance limits in a pre-sleep state.

**Table 4:** Comparison of tolerance limits for different sound variables.

Sound	Tolerance limit average value (dB)	Standard deviation	*p* Value
Traffic noise	38.018	3.1461	0.000
Birdsong	37.179	3.2353	0.033
Conversation voice	34.786	1.7290	0.002

The experimental results show that the minimum tolerance of the voice of the participants is lower than that of traffic noise and birdsong, which is consistent with practical experience. On the one hand, the frequency range of human voice is 300 ~ 3,400 Hz, while the bandwidth of music, wind and rain, car and other sounds is wider, which can reach 20 Hz ~ 20 kHz, which is often defined as white noise with recovery effect (Hong et al., 2020); On the other hand, there is too much information in the vocal cords that people can understand (Tvr et al., 2020). Therefore, the participants are more sensitive to the voice and have a very low minimum tolerance for the voice.

### 3.2. Single-variable test results of light pollution

The analysis of variance (ANOVA) and homogeneity test of variance of the univariate strong light endurance test results showed that there was no significant difference in the tolerance limits of participants to different internal light variables (*p* = 0.288, *p* = 0.122, *p* = 0.146). The ANOVA showed that there was no significant difference in the effect of unilateral light (2,700 K) on the tolerance limits of participants (*p* = 0.288 > 0.05). Furthermore, there was no significant difference in the effect of unilateral light (4,000 K; *p* = 0.122 > 0.05), and there was no significant difference in the effect of unilateral light (6,500 K; *p* = 0.146 > 0.05; Table 5). The results showed that the evaluation values of obtrusive light all exceeded 280 l ×, which is the upper limit of the average illuminance of the vertical of window external surfaces. This means that, under the condition of 280 l × average window illuminance, the participants did not have the feeling of light invasion regarding the light variable of the three color temperatures. This result is quite different from that of Sim et al. (2016). In their study, when the average illuminance of the vertical of a window’s external surface reached 70 lx, the disturbing effect was significant. After further analyzing the two studies (Chan, 2021), experiment was conducted in a pure laboratory environment, while the experiment conducted in this study was closer to a real-life environment. Under the condition of frosted glass, the indoor side view window is more like a luminous light box. Through frosted glass, light enters the room and illuminates every interface of the living environment, which may make the participant feel that he or she is living in an environment with ordinary light.

**Table 5:** Comparison of tolerance limits for different light variables.

Light	Tolerance limit average value (DGInght Index)	Standard deviation	Average vertical illuminance outside window (DGInght Index)	Maximum luminance of inner surface of window (cd/m^2^)
Fixed light (2,700 K)	21.52	2.53	5.43	93.91
Fixed light (4,000 K)	21.09	2.79	5.57	93.91
Fixed light (6,500 K)	20.67	2.80	5.70	93.91

These two experimental environments might be the main reason for the significant difference between the two results (Table 6).

**Table 6:** Test of homogeneity of variances.

		Levene Statistic	DF 1	DF 2	Sig.
Fixed light (2,700 K) Testing sound (Traffic)	Based on mean	0.604	1	26	0.444
Fixed light (2,700 K) Testing sound (Conversation)	Based on mean	2.129	1	26	0.156
Fixed light (2,700 K) Testing sound(Birdsong)	Based on mean	0.353	1	26	0.557
Fixed light (4,000 K) Testing sound (Traffic)	Based on mean	1.646	1	26	0.211
Fixed light (2,700 K) Testing sound(Birdsong)	Based on mean	4.964	1	26	0.035
Fixed light (4,000 K) Testing sound (Birdsong)	Based on mean	0.315	1	26	0.579
Fixed light (4,000 K) Testing sound (Traffic)	Based on mean	0.417	1	26	0.524
Fixed light (6,500 K) Testing sound (Conversation)	Based on mean	1.124	1	26	0.299
Fixed light (4,000 K) Testing sound (Conversation)	Based on mean	0.602	1	26	0.445

### 3.3. Adding sound pollution to light pollution at tolerance limit

ANOVA showed that the tolerance limit of sound intrusion if the tolerance limit of the participants to light intrusion fixed was no significant. The results of ANOVA analysis are summarized in Table 7.

**Table 7:** ANOVA of fixed light adding sound on the tolerance limit of participants.

ANOVA						
		SS	DF	MS	F	Significance
Fixed light (2,700 K) Testing sound (Traffic)	Factor	10.374	1	10.374	1.214	0.281
	Error	222.126	26	8.543		
Fixed light (2,700 K) Testing sound (Conversation)	Factor	9.689	1	9.689	3.168	0.087
	Error	79.526	26	3.059		
Fixed light (2,700 K) Testing sound (Birdsong)	Factor	0.119	1	0.119	0.012	0.914
	Error	261.908	26	10.073		
Fixed light (4,000 K) Testing sound (Traffic)	Factor	5.836	1	5.836	1.314	0.262
	Error	115.477	26	4.441		
Fixed light (4,000 K) Testing sound (Conversation)	Factor	5.803	1	5.803	2.063	0.163
	Error	73.126	26	2.813		
Fixed light (4,000 K) Testing sound (Birdsong)	Factor	6.577	1	6.577	0.716	0.405
	Error	238.664	26	9.179		
Fixed light (6,500 K) Testing sound (Traffic)	Factor	10.859	1	10.859	2.290	0.142
	Error	123.310	26	4.743		
Fixed light (6,500 K) Testing sound (Conversation)	Factor	7.072	1	7.072	3.363	0.078
	Error	54.669	26	2.103		
Fixed light (6,500 K) Testing sound (Birdsong)	Factor	1.262	1	1.262	0.156	0.696
	Error	210.703	26	8.104		

SS=Sum of squares; DF=Degree of freedom, MS = Mean square; Sig = Significance.

**Figure 5:**
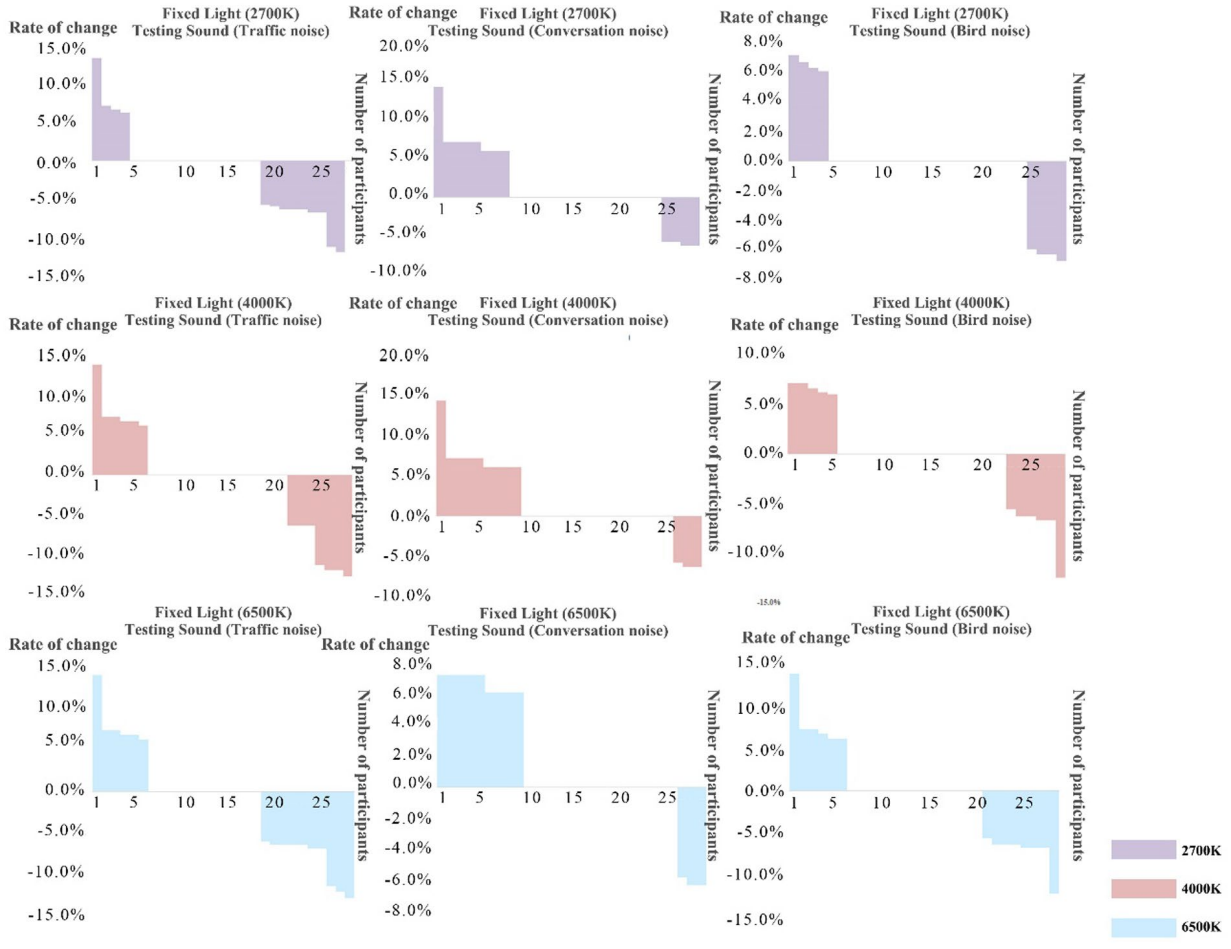
The percentage between the tolerance limit of sound–light interaction and a single sound intrusion is in descending order before and after interaction.

This study ranked the percentages presented in descending order by comparing the tolerance limits Bi of participants to sound interference after univariate interaction with sound interference. Based on the observations in Figure 5, the decrease in endurance after intrusion interaction was less. Therefore, the limit of endurance of light intrusion was not significantly reduced due to the interaction of variables.

**Figure 6:**
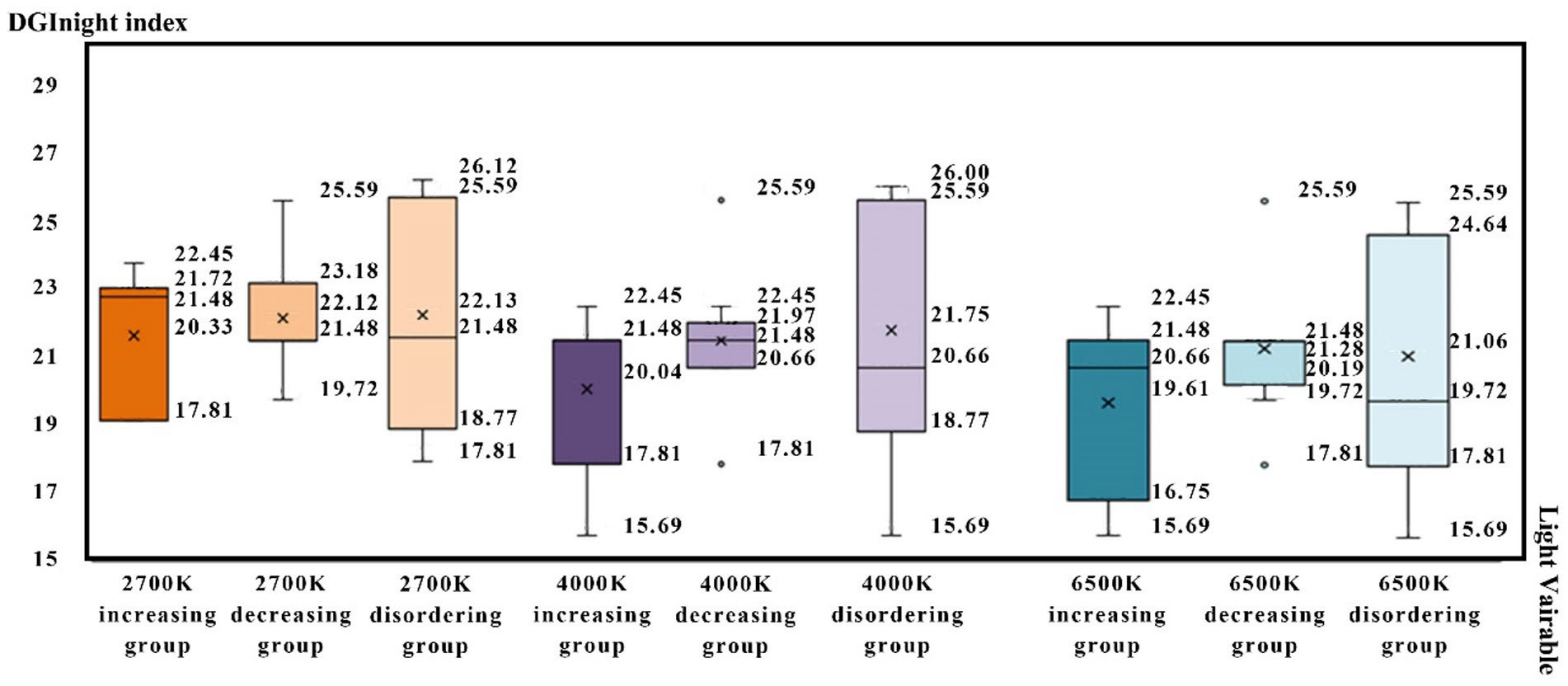
Characteristic box diagram of tolerance limits distribution of the participants under different color, temperature, and light disturbances.

Therefore, based on the observations in Figure 6, the lower the comprehensive tolerance limits of sound intrusion of a participant, the higher the probability that their tolerance limits will increase after sound–light interaction.

This study calculated and compared the average value of sound intrusion tolerance limits of all participants and three groups of participants to the three different sound types. The researchers observed that the average value of univariate tolerance limits of the “increase group” was lower than that of all participants, while the results for the “decrease group” and “disorder group” were contrary. In Figure 5, the lower the comprehensive tolerance limit of the participant’s sound intrusion, the higher the probability that the tolerance limits will increase after sound–light interaction.

### 3.4. Adding light pollution to sound pollution at tolerance limits

**Figure 7:**
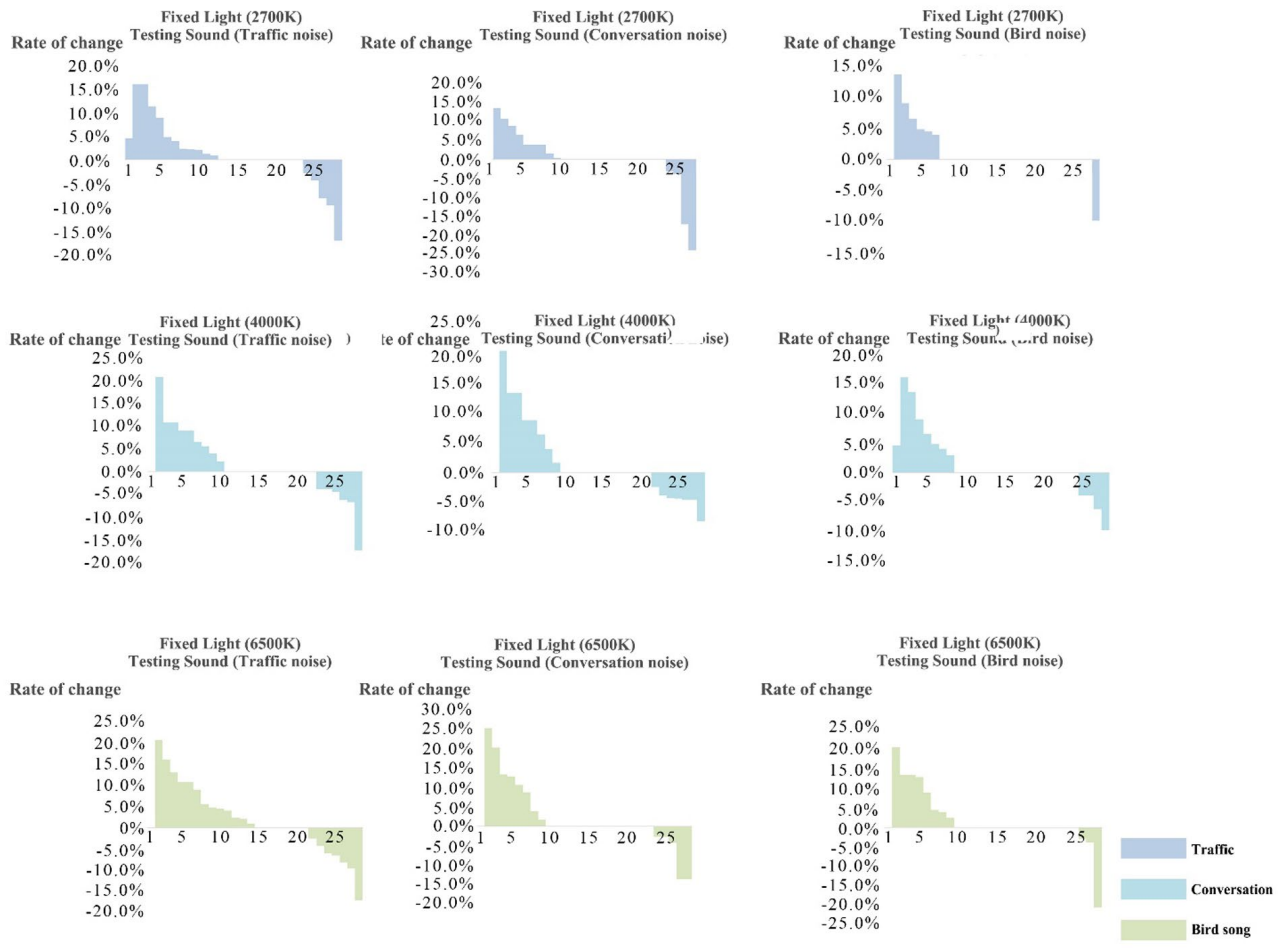
Descending order in percentage of comparison between tolerance limit to light intrusion and the single variable of obtrusive light before and after interaction.

From Figure 7, it can be seen that the decrease in tolerance after intrusion interaction is relatively small. So, the tolerance limits of light intrusion would not be significantly reduced due to interaction of variables. The lower the tolerance limits to sound intrusion, the higher the possibility that the tolerance limits improved after sound–light interaction.

**Figure 8:**
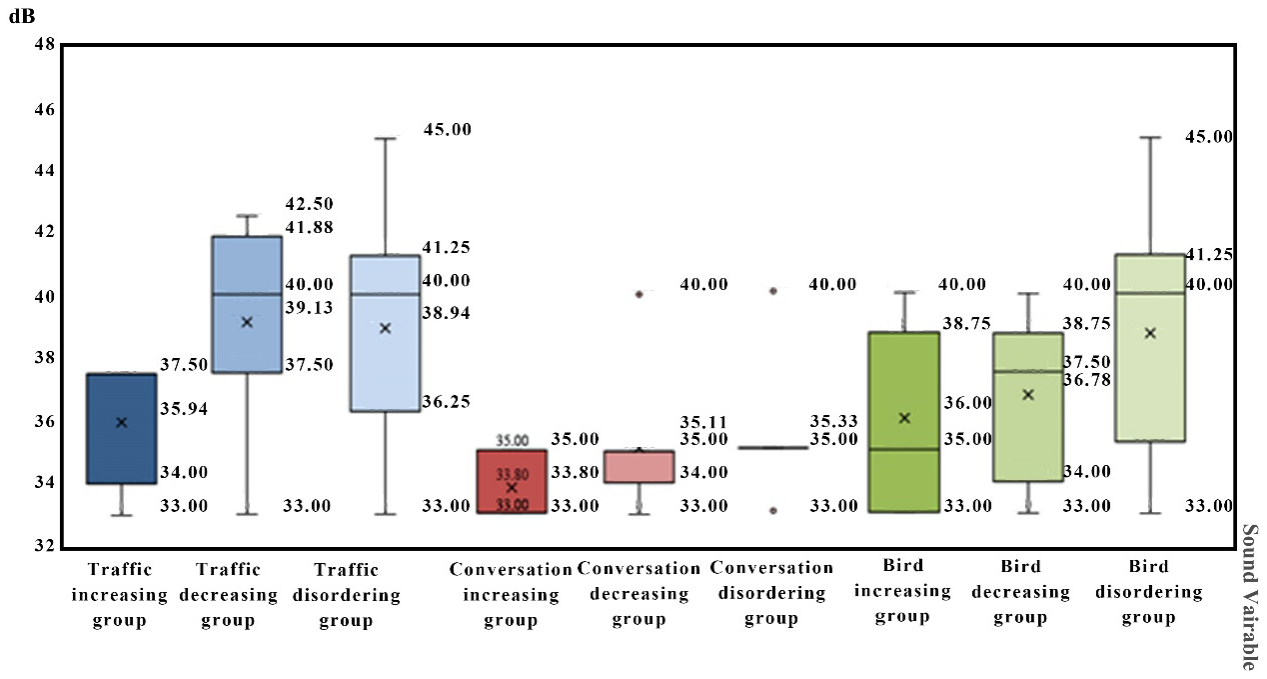
Characteristic box diagram of tolerance limits distribution of participants under different sound disturbances.

From Figure 8, it can be seen that the decrease in tolerance after intrusion interaction is relatively small. So, the tolerance limits of sound intrusion not be significantly reduced due to interaction of variables. The lower the comprehensive tolerance limits of participants to light intrusion, the higher the possibility that the tolerance limit improved after sound–light interaction.

The results of ANOVA showed that there was no significant difference in the tolerance limits of light intrusion if the tolerance limit of the participants to the sound intrusion was fixed.

The researchers sorted, in descending order, the percentages by comparing the tolerance limits of the participants to light intrusion after interaction with the light intrusion single variable. Based on the observations in Figure 6, the number of decreases in endurance after intrusion interaction was less. Therefore, the limit of endurance of sound intrusion will not be significantly reduced due to interaction of variables.

In the same way, the results from the tolerance limit test were divided into three groups: increasing tolerance limit, decreasing tolerance limit, and disordered tolerance limit, according to the difference in mutual tolerance limits between single variables and variable interaction. The results are as follows.

Participants whose tolerance limits increased but did not decrease after interaction of variables were classified as the group with increasing tolerance limit; after testing, there were 15 participants in this group.

Participants whose tolerance limits decreased but did not increase after interaction of variables were classified as the group with decreasing tolerance limit; after testing, there were eight participants in this group.

Participants whose tolerance limits both decreased and increased after interaction of variables were classified as the group with disordered tolerance limit; after testing, there were five participants in this group.

**Figure 9:**
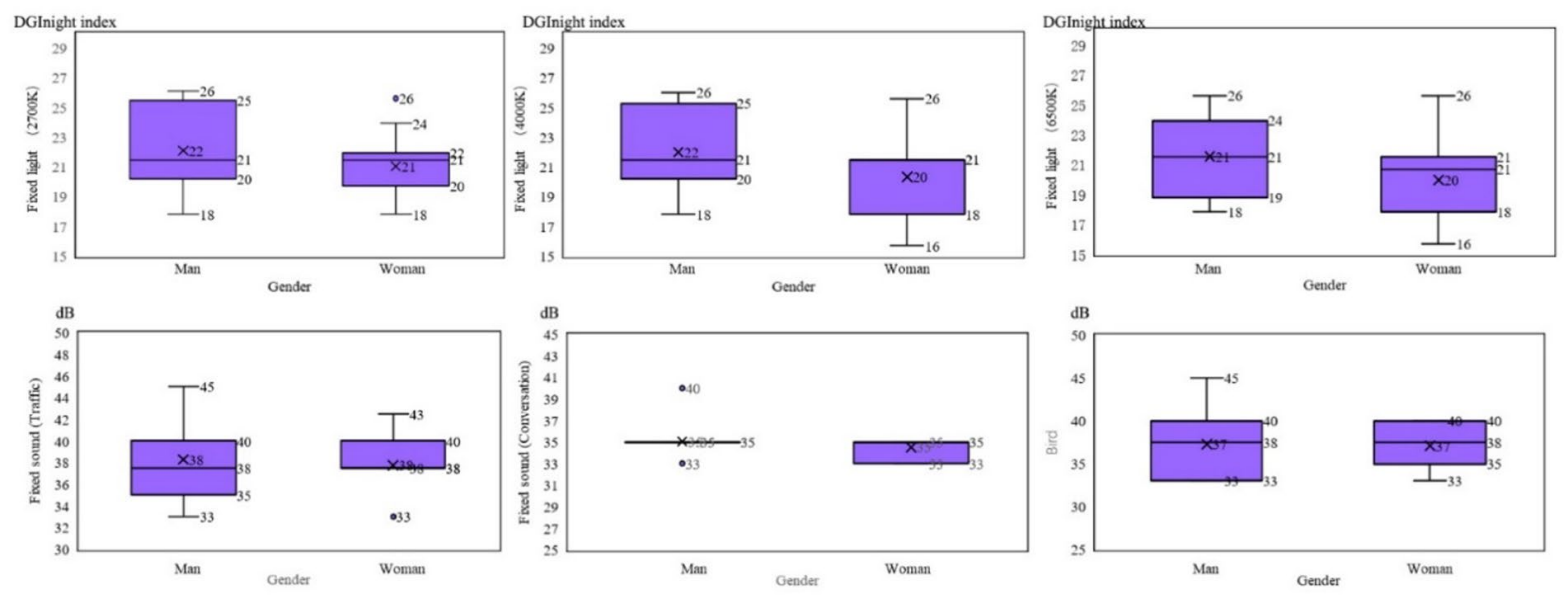
Box diagram of different genders under sound–light interaction.

By counting and comparing the tolerance limits average value of light intrusion of all the participants and the three groups of participants to three different color temperatures, it was found that the tolerance limits average value of the single variable in the “increasing group” was lower than those of all participants, while the results in the “decreasing group” and “disordered group” were contrary. Therefore, based on Figure 9, the lower the tolerance limits of light intrusion of a participant, the higher the probability that their tolerance limits will increase after sound–light interaction.

However, on the basis of fixed sound intrusion, increasing the single metering light with different color temperatures will impact the participants’ tolerance limits. Specifically, the single metering light with one-sided light 2,700 K (*F*(1,26) = 0.414, *p* = 0.032 < 0.05) and one-sided light 4,000 K (F(1,26) = 0.784, *p* = 0.015 < 0.05) color temperature significantly reduced the sound pressure level (dB) value of the participant, reaching the tolerance limit when applied to traffic sound intrusion. The single metering light with 6,500 K color temperature (F(1,26) = 0.840, *p* = 0.08 < 0.05) has no effect on traffic sound intrusion. For human voice, application of 2,700 K single light metering has effect on the tolerance limit of participants under human voice intrusion(F(1,26) = 0.204, *p* = 0.07 < 0.05), when application of 4,000 K single light metering (F(1,26) = 0.446, *p* = 0.030 < 0.05) and 6,500 K single light metering (F(1,26) = 0.835, *p* = 0.036 < 0.05) significantly increases the dB value of participants reaching the tolerance limits. On the basis of birdsong intrusion, increasing the single photometry of different color temperatures had a significant impact on participants’ tolerance limits, which is specifically reflected in the increase of 2,700 K single side light (F(1,26) = 0.784, *p* = 0.015 < 0.05), 4,000 K single side light (F(1,26) = 0.088, *p* = 0.046 < 0.05), and 6,500 K single side light (F(1,26) = 0.088, *p* = 0.026 < 0.05; see Tables 8, 9).

**Table 8:** Test of homogeneity of variances.

	Levene Statistic	DF 1	DF 2	Sig.	
Fixed sound (Traffic) Testing light (2,700 K)	Based on Mean	0.689	1	26	0.414
Fixed sound (Traffic) Testing light (4,000 K)	Based on Mean	0.076	1	26	0.784
Fixed sound (Traffic) Testing light (6,500 K)	Based on Mean	0.042	1	26	0.840
Fixed sound (conversation) Testing light (2,700 K)	Based on Mean	1.701	1	26	0.204
Fixed sound (conversation) Testing light (4,000 K)	Based on Mean	0.599	1	26	0.446
Fixed sound (conversation) Testing light (6,500 K)	Based on Mean	0.044	1	26	0.835
Fixed sound (Birdsong) Testing light (2,700 K)	Based on Mean	1.616	1	26	0.215
Fixed sound (Birdsong) Testing light (4,000 K)	Based on Mean	3.136	1	26	0.088
Fixed sound (Birdsong) Testing light (6,500 K)	Based on Mean	0.007	1	26	0.934

**Table 9:** ANOVA of the impact of light pollution on the endurance limit of different sex groups on the basis of sound pollution.

		SS	DF	MS	*F*	Significance
Fixed sound (Traffic) Testing light (2,700 K)	Factor	30.512	1	30.512	5.110	0.032
	Error	155.254	26	5.971		
Fixed sound (Traffic) Testing light (4,000 K)	Factor	47.980	1	47.980	6.856	0.015
	Error	181.967	26	6.999		
Fixed sound (Traffic) Testing light (6,500 K)	Factor	24.359	1	24.359	3.313	0.080
	Error	191.163	26	7.352		
Fixed sound (conversation) Testing light (2,700 K)	Factor	17.922	1	17.922	3.559	0.070
	Error	130.916	26	5.035		
Fixed sound (conversation) Testing light (4,000 K)	Factor	27.076	1	27.076	5.299	0.030
	Error	132.855	26	5.110		
Fixed sound (conversation) Testing light (6,500 K)	Factor	31.788	1	31.788	4.897	0.036
	Error	168.777	26	6.491		
Fixed sound (Birdsong) Testing light (2,700 K)	Factor	27.814	1	27.814	5.396	0.028
	Error	134.024	26	5.155		
Fixed sound (Birdsong) Testing light (4,000 K)	Factor	24.565	1	24.565	4.372	0.046
	Error	146.086	26	5.619		
Fixed sound (Birdsong) Testing light (6,500 K)	Factor	37.624	1	37.624	5.574	0.026
	Error	175.503	26	6.750		

SS=Sum of squares; DF=Degree of freedom, MS = Mean square; Sig = Significance.

### 3.5. Influence of sound–light interaction on tolerance limit of participants

According to the Chi-square test of 24 experiments on the basis of fixed single photometry, whether it is 2,700 K single photometry, 4,000 K single photometry, or 6,500 K single photometry, the increase in sound intrusion has no clear impact on the tolerance limit of participants.

On the basis of the above findings, this paper further analyzes the different characteristics of men’s and women’s tolerance under light disturbance and sound disturbance through the contingency table method of analysis.

Figure 8, the box diagram, shows the comparison of mean values, it can be seen that under the interference of one-sided light at three color temperatures, the DGI attaining the tolerance limit of men is greater than that of women. For acoustic intrusion, the decibel values for men reaching the tolerance limit under interference from the three types of noise are similar to that of women (Table 10).

**Table 10:** Chi-square test.

	Pearson x2	*k*	sig
Fixed light (2,700K)	26.286^a^	9	0.002
Fixed light (4,000K)	15.714^b^	8	0.047
Fixed light (6,500K)	15.714^b^	8	0.047
Traffic	11.429^c^	5	0.044
Conversation	14.000^d^	2	0.001
Birdsong	12.714^c^	5	0.026
Fixed sound (Traffic) Testing light (2,700K)	27.714^e^	11	0.004
Fixed sound (Traffic) Testing light (4,000K)	31.286^a^	9	0.000
Fixed sound (Traffic) Testing light (6,500K)	28.571^b^	8	0.000
Fixed sound (conversation) Testing light (2,700K)	29.857^b^	8	0.000
Fixed sound (conversation) Testing light (4,000K)	26.857^f^	7	0.000
Fixed sound (conversation) Testing light (6,500K)	14.429^b^	8	0.071
Fixed sound (Bird song) Testing light (2,700K)	29.143^a^	9	0.001
Fixed sound (Bird song) Testing light (4,000K)	33.714^f^	7	0.000
Fixed sound (Bird song) Testing light (6,500K)	29.357^g^	10	0.001
Fixed light (2,700K) Testing sound (Traffic)	7.714^h^	4	0.103
Fixed light (2,700K) Testing sound (conversation)	12.286^i^	3	0.006
Fixed light (2,700K) Testing sound (Bird song)	12.714^c^	4	0.026
Fixed light (4,000K) Testing sound (Traffic)	26.643^h^	5	0.000
Fixed light (4,000K) Testing sound (conversation)	17.429^i^	4	0.001	Fixed light (4,000K) Testing sound (Bird song)	5.214^h^	4	0.266	Fixed light (6,500K) Testing sound (Traffic)	18.786^h^	4	0.001	Fixed light (6,500K) Testing sound (conversation)	7.357^d^	2	0.025
Fixed light (6,500K) Testing sound (Bird song)	5.929^h^	4	0.205

^a^The expected frequency for 10 cells (100.0%) is less than 5. The expected minimum cell frequency is 2.8. ^b^The expected frequency of 9 cells (100.0%) is less than 5. The expected minimum cell frequency is 3.1.^c^The expected frequency of 6 cells (100.0%) is less than 5. The expected minimum cell frequency is 4.7.^d^The expected frequency of 0 cells (0.0%) is less than 5. The expected minimum cell frequency is 9.3.^e^The expected frequency of 12 cells (100.0%) is less than 5. The expected minimum cell frequency is 2.3.^f^The expected frequency of 8 cells (100.0%) is less than 5. The expected minimum cell frequency is 3.5.^g^The expected frequency of 11 cells (100.0%) is below 5. The expected minimum cell frequency is 2.5.^h^The expected frequency of 0 cells (0.0%) is less than 5. The expected minimum cell frequency is 5.6.^i^The expected frequency of 0 cells (0.0%) is less than 5. The expected minimum cell frequency is 7.0.

It can be seen from the above table that the Chi-square test is used to study whether the differences between the gender of the subjects on acoustic pollution, light pollution and sound–light interactive pollution are significant. It can be seen that the significance of 24 kinds of sound–light interactive pollution is greater than 0.05, which means that there is no significant difference in the tolerance limit of participants of different sexes to different color temperatures, different noise and sound–light interaction pollution.

## 4. Discussion

### 4.1. In comparison with the combined effects of environmental variables.

A large number of sample questionnaires have shown that visual elements can change individual voice perception. Renterghem and Botteldooren (2016) showed that when the vegetation was completely invisible, residents had a 34% chance of being moderately disturbed by noise, while for the respondents with very obvious vegetation view, the proportion decreased to 8%. In this study, we discussed the weakening effect of light with different color temperatures on the noise in the urban physical environment: the research conclusion shows that adding light with different color temperatures on the basis of human voice can improve the endurance limit of participants to human voice (1–2%), which is similar to the research conclusion of Van Ranterghem and Botteldooren. Ren (2023) showed that the dominant sound (different sound types) will significantly affect the acoustic comfort of visitors in urban open space through their research on the acoustic comfort of visitors. In this study, the sound of birds and traffic will reduce the endurance limit of participants to different color temperature light, which is consistent with Xinxin Ren’s research.

### 4.2. Design strategy

**Figure 10:**
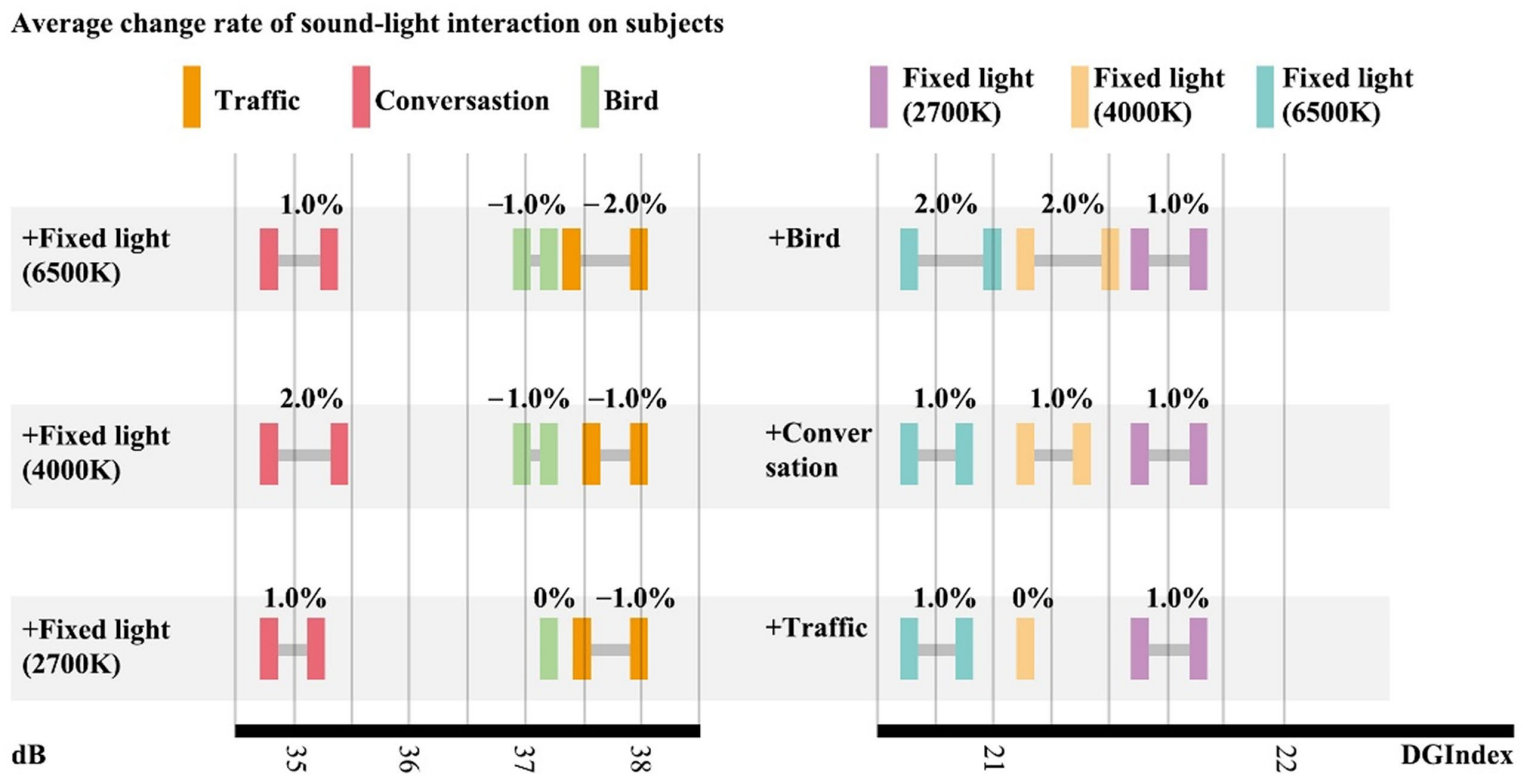
Portrait of the influence of sound–light interaction on participants.

Through collation of the experimental data, the effects of superimposed pollution relative to single pollution. The participants are divided mainly along two aspects, namely, the influence of the tolerance limit of the participant under light intrusion and the tolerance limit of the participant under sound intrusion. By analyzing and comparing the changes in the tolerance limits of participants under different light intrusions and a single sound variable under fixed light conditions, and the changes in the tolerance limits of participants under different sound intrusions and a single light variable under fixed light conditions, the individual differences of the influence of combined sound and light become clear. Note that the participant portrait (Figure 10) contradicts the results from intra-individual differences.

In this study, the impact of sound environment on endurance limit is stronger than that of light environment. This finding has been supported by previous studies by some scholars, such as Jiang et al. (2021) and others through laboratory simulation research found that the acoustic environment has a stronger impact on human multi-dimensional emotional state than the visual environment. Hui and Shan (2018) and others found that the acoustic environment has a greater impact on the mental recovery of office workers than the visual environment. Based on the above research and conclusions, it provides a theoretical basis for the introduction of soundscape creation and design strategies to create natural healing effects when building a healthy city in the field of urban planning.

Through the analysis of difference within the sample under the interaction of sound and light, on the basis of fixed one-sided light, regardless of the color temperature, increasing different sound intrusions has no clear impact on tolerance limits. Through the analysis of difference between samples of sound–light interaction, for the human voice and birdsong, the impact can be mitigated *via* application of light. For traffic noise, simultaneous intrusion of light pollution should be avoided.

### 4.3. Limitation

After adding another kind of intrusion, some participants’ tolerance increased, while others’ decreased. This difference from noise annoyance depends on two factors, the noise source and participants’ personality traits. Melika et al. (2020) and other scholars have studied the relationship between individual participant characteristics and noise tolerance and found that extroversion and neuroticism are most important in noise sensitivity and annoyance, while responsibility and openness to experience are considered the least important variables. In this study, the change of tolerance in different directions after intrusion superposition can be speculated as being closely related to individual moral and behavioral characteristics.

In analyzing individual differences in the influence of sound–light interaction on tolerance limit, the individual reporting method is adopted, which lacks physiological testing of participants, such as an EEG or dermatogram. Recently, many studies have used these methods to generate evidence for investigating neural activity and emotion (Chan, 2021). In addition, there is a lack of tests on psychological characteristics, such as reaction ability, personality, and mental health level, including the Big Five personality traits. This has been shown to affect the current state of fatigue, arousal, and emotion (Knez, 1995; Zhang et al., 2018). Yang et al. (2022) and Burattini et al. (2019) can quantitatively assist in determining the tolerance limits and can provide psychological explanations. However, the researchers have not identified the personality traits (Liebl et al., 2012) that affect the tolerance limits. Future work is expected to reveal the influence of personality traits on tolerance limit tolerance limits through rigorous psychological and physiological testing of participants.

## 5. Conclusion

Sound–light pollution is an environmental problem that affects the quality of human life and endangers health. Previous studies have focused on the multi-sensory interaction between office space (Jeon et al., 2022) or urban public space (Meihui et al., 2020). On the one hand, and the interaction between positive sound and positive vision on the other hand. For example, Jiang et al. (2021) has studied the interaction between bird song and outdoor landscape. This paper examines a cross sound–light sensory channel to study the interaction of sound–light pollution in urban residential environments at night. This study found that the tolerance limit of participants was not reduced due to superposition of two intrusive variables.

There were significant differences in the tolerance limit of participants to different content sound variables. Among them, the traffic noise has the greatest impact on the tolerance limits of people, followed by birdsong and human voice.

There were no significant differences in the tolerance limit of participants to light variables at different color temperatures The results showed that the tolerance limit exceeded 23.99 DGInight Index. Luminance is the main factor affecting glare, and the influence of color temperature is not significant.

Adding light pollution to sound pollution can increase the tolerance limits of participants, while adding sound pollution to light pollution has no significant effect on the tolerance limits. The physiological and psychological differences between participants may affect the performance differences of individual participants in the interaction between sound and

## Data availability statement

The raw data supporting the conclusions of this article will be made available by the authors, without undue reservation.

## Ethics statement

The studies involving human participants were reviewed and approved by IRB Tsinghua University. The patients/participants provided their written informed consent to participate in this study.

## Author contributions

YY: conceptualization, investigation, methodology, formal analysis, and writing—original draft. DF: data curation and visualization. JK: writing—review and editing, and supervision. XZ: funding acquisition, project administration, supervision, resources, conceptualization, and writing—review and editing. All authors contributed to the article and approved the submitted version.

## Funding

This work was supported by the National Natural Science Foundation of China (no. 52078266) and Tsinghua University Initiative Scientific Research Program (no. 20211080095).

## Conflict of interest

The authors declare that the research was conducted in the absence of any commercial or financial relationships that could be construed as a potential conflict of interest.

## Publisher’s note

All claims expressed in this article are solely those of the authors and do not necessarily represent those of their affiliated organizations, or those of the publisher, the editors and the reviewers. Any product that may be evaluated in this article, or claim that may be made by its manufacturer, is not guaranteed or endorsed by the publisher.
